# Circulating Antibody and Memory B-Cell Responses to *C. difficile* Toxins A and B in Patients with *C. difficile*-Associated Diarrhoea, Inflammatory Bowel Disease and Cystic Fibrosis

**DOI:** 10.1371/journal.pone.0074452

**Published:** 2013-09-10

**Authors:** Tanya M. Monaghan, Adrian Robins, Alan Knox, Herbert F. Sewell, Yashwant R. Mahida

**Affiliations:** 1 Institute of Infection, Immunity and Inflammation, University of Nottingham and Nottingham University Hospitals NHS Trust, Nottingham, United Kingdom; 2 Division of Immunology, University of Nottingham, Nottingham, United Kingdom; 3 Division of Respiratory Medicine, University of Nottingham and Nottingham University Hospitals NHS Trust, Nottingham, United Kingdom; University of Leicester, United Kingdom; Wayne State University, United States of America

## Abstract

*C. difficile* infection (CDI) is rarely reported in cystic fibrosis (CF) patients despite frequent hospitalisations and antibiotic usage. Conversely, the prevalence of CDI in inflammatory bowel disease (IBD) has received increased attention. We investigated components of the IgG-specific humoral immune response to *C. difficile* toxins A and B in patients with *C. difficile*-associated diarrhoea (CDAD), IBD patients with CDI, CF patients and healthy controls. Serum anti-toxin IgG was determined by ELISA. Circulating antigen-activated B-cells were investigated using Alexa Fluor 488-labelled toxin A and assessed by flow cytometry. Following induction of differentiation of memory B-cells, toxin A- and B-specific antibody secreting cells (ASCs) were quantified using ELISpot. We present the first data showing levels of serum anti-toxin A and B antibodies were significantly higher in patients with CF (without a history of CDI) than in CDAD patients and were stably maintained over time. Notably, the CDAD patients were significantly older than the CF patients. We also show that circulating toxin A-specific memory B-cells (IgD-negative) can be detected in CDAD patients [0.92 (0.09–1.78)%], and were prominent (5.64%, 1.14%) in two CF patients who were asymptomatic carriers of *C. difficile*. There was correlation between toxin A- and B-specific ASCs, with significantly higher proportions of the latter seen. In some with CDAD, high serum antibody levels were seen to only one of the two toxins. Mucosal secretion of toxin-specific IgG was detected in an additional group of IBD patients with no history of CDI. We conclude that enhanced and stable humoral immune responses to toxins A and B may protect CF and some IBD patients against CDI. The impaired ability to generate strong and/or sustained toxin-specific antibody and memory B-cell responses may increase susceptibility of older patients to CDI and highlight the need to investigate the role of immune senescence in future studies.

## Introduction

*C. difficile* infection is a significant clinical problem in patients exposed to the hospital environment. Clinical presentation in those colonised with toxigenic *C. difficile* can include asymptomatic carriage, mild self-limiting diarrhoea and severe life-threatening pseudomembranous colitis. Exposure to antibiotics is the commonest risk factor, facilitating colonization by *C. difficile*, via disruption of the protective resident microflora [1,2,3].

Host factors that may determine the development and nature of clinical disease are not fully understood but include age, the use of proton-pump inhibitors or H2 blockers, tube feeding and the host immune response [1,3,4,5,6]. Secreted toxins A and B mediate the intestinal disease [1,7] and serum IgG antibodies against toxin A appear to dominate protective responses in humans; more recently, antitoxin B responses have been correlated with colonization [3] and prevention of recurrent disease [8]. There are also recent reports of an increase in susceptibility to *C. difficile* infection in patients with inflammatory bowel disease [9]. By contrast, *C. difficile*-associated disease is uncommon in patients with cystic fibrosis, despite frequent admissions to hospital for antibiotics for acute pulmonary exacerbations. Asymptomatic carriage of *C. difficile* has been reported in 22-32% of such patients [10,11], but the mechanism of protection against the development of colonic disease remains to be determined.

The aim of our studies was to investigate, directly for the first time, B lymphocyte anti-toxin A and anti-toxin B antibody production as well as IgG-specific humoral immune responses in patients with *C. difficile*-associated diarrhoea, *C. difficile* infection in patients with inflammatory bowel disease and patients with cystic fibrosis.

## Materials & Methods

### Subjects

Blood samples were obtained from adult healthy donors (19) and patients attending two major hospitals in Nottingham, UK between June 2009 and April 2012 (34 months). They included: (i) 53 patients with *C. difficile*-associated diarrhoea, (ii) 10 inflammatory bowel disease (7 ulcerative colitis, 3 Crohn’s disease) patients with *C. difficile* infection and (iii) 18 patients with cystic fibrosis. The diagnosis of cystic fibrosis had previously been made on the basis of a positive sweat test and/or demonstration of 2 known cystic fibrosis mutations and typical clinical features of the disease. Intestinal mucosal samples from an additional 15 patients with inflammatory bowel disease (without a history of *C. difficile* infection) were also studied.

Written informed consent, specific for each sample type (blood, stool, mucosal tissue), was obtained before collection. These studies were approved by the Nottingham Research Ethics Committee, which also approved the consent procedure for each sample type.

All the patients with *C. difficile* infection had diarrhoea (defined as a change in bowel habit with 3 or more unformed stools per day for at least 48 hours) and positive stool *C. difficile* toxin test. Asymptomatic carriers were defined as those without diarrhoea, but had a positive stool culture for *C. difficile*.

### Sample collection

Serum was separated from venous blood samples and aliquots stored at -80^0^C, until use in ELISA assays. Peripheral blood mononuclear cells (PBMCs) were isolated by centrifugation of EDTA-treated venous blood samples over a Ficoll-Hypaque (Sigma) density gradient. These cells were subsequently used to study memory B cells and toxin A-activated B cells. Frozen stool samples were treated and cultured for *C. difficile*, as previously described [12]. Mucosal samples were obtained from surgically-resected intestinal tissue of patients with inflammatory bowel disease. Following removal of epithelial cells, mucosal samples were cultured, as previously described [13]. The presence of toxin-specific IgG antibodies in supernatant samples was subsequently investigated by ELISA.

### Memory B cell stimulation assay

For induction of differentiation of memory B cells to antibody secreting cells, the peripheral blood mononuclear cells were cultured (0.5 x 10^6^ cells/ml, at 37^0^C, in 5% CO_2_) for 6 days in RPMI (supplemented with 2 mM L-glutamine), 10% fetal calf serum, 0.1 mg/ml penicillin G (Britannia Pharmaceuticals, UK), 0.1 mg/ml streptomycin (Sigma-Aldrich), and 0.1 mg/ml gentamicin (Mayne Pharma Plc, UK), type B CpG oligonucleotide (ODN 2006; 6 µg/ml; InvivoGen), recombinant human interleukin (IL)-21 (Miltenyi Biotech) and recombinant human CD40 ligand (Miltenyi Biotech). Following centrifugation, aliquots of culture supernatant samples were stored at -80^0^C. After washing, the cells were used in ELISpot assays.

### Flow cytometry staining and analysis

Antigen-activated B cells were investigated using Alexa Fluor^®^ 488-labelled toxin A (toxin A^488^ [14]). Peripheral blood mononuclear cells (2 x 10^6^) were incubated on ice with 10 µg/ml toxin A^488^ on a rocker, in the dark, for 1 hour. After washing, the cells were labelled with antibodies to CD19 (Beckman Coulter) and IgD (Southern Biotech). Washed cells were fixed in 0.5ml of 0.5% formaldehyde and subsequently studied by flow cytometry (FACScan FC500, Beckman-Coulter). Data files of up to 1x10^6^ events were collected for each test sample and the resultant dot-plots were opened in Weasel version 3 software (Walter and Eliza Hall Institute of Medical Research) which was used to draw regions encompassing toxin A^488^ positively labelled events.

### ELISA

Toxin A- and toxin B-specific IgG responses in serum samples were determined by ELISA. Flat-bottom 96-well ELISA plates (F96 CERT Maxisorp NUNC, Thermo Scientific) were coated with 100µl of a 30µg/ml solution of purified toxin A or B in 0.05 M carbonate-bicarbonate buffer (pH 9.6) and incubated overnight at 4°C. After washing six times with PBS-0.05% Tween 20 using an automatic plate washer (ELx50 automated strip washer; Biotek Instruments, Inc.), blocking solution (PBS-0.05% Tween 20 containing 0.05% azide and 1% goat serum) was added to each well and incubated for three hours at 25°C. After further washing, sera diluted (1:100) in PBS-0.05% Tween 20 containing 0.05% azide (or lamina propria cell culture supernatant samples at 1:4 dilution) were added to each well (100 µl) in triplicate and incubated for two hours at 25°C. After washing, positive reactions were detected by successive incubations with 100µl dilution of 5 µg/ml biotinylated anti-human IgG (Vector) in PBS-0.05% Tween 20 containing 1% goat serum for one hour at 25°C and with a streptavidin-horseradish peroxidase substrate (Vector) for 20 minutes at 25°C. The final reaction was visualized by the addition of 2,2’-azino-bis (3-ethylbenzthiazoline-6-sulfonic acid) for 20 minutes in the dark. *A*_405_ values were measured in an ELISA plate reader. The *A*_405_ values for the negative control wells were averaged and subtracted from toxin-coated wells to give the corrected *A*_405_ values for each test sample. ELISA units (EU) in test sera were then calculated from their OD_405_ values using a standard curve established from serial dilutions (1:25 to 1:1600) of pooled serum samples with high anti-toxin antibody titres. If the corrected OD_405_ value of a sample fell below that of the lower plateau in the standard curve, it was deemed negative and given an arbitrary value of 0.01 EU.

### ELISpot

Ninety six-well ELISpot plates (Millipore Multiscreen MSIPS4510 HTS IP Sterile plate 0.45 µm, hydrophobic, high-protein binding) were pre-treated with 15 µl of 35% ethanol for 1 minute. After three washes with PBS the plates were incubated overnight (at 4^°^C) with goat anti-human IgG Fc fragment (10 µg/ml, from Bethyl Laboratories, to detect all IgG-secreting cells) and either PBS (control), purified toxin A or purified toxin B (both at a concentration of 30 µg/ml). After washing 4 times with PBS, plates were blocked with 200µl/well of RPMI-1640 supplemented with 10% FCS. To induce differentiation of memory B cells to antibody secreting cells, mitogen-stimulated peripheral blood mononuclear cells were subsequently applied to ELISpot plates (25 x 10^3^ cells/well to detect total IgG ASCs or 2 x 10^5^ cells/well to detect toxin-specific ASCs). Control wells that were incorporated in each plate included those with no cells (media only), and wells to which unstimulated peripheral blood mononuclear cells were added. Following incubation for 18 hours (at 37^°^C, 5% CO_2_), the wells were washed 4 times with PBS, followed by PBS-0.05% Tween 20. Diluted (1:1000 in PBS-0.05% Tween 20) horseradish peroxidise-conjugated goat anti-human IgG Fc fragment antibodies (Jackson Immunoresearch) were added to the wells and incubated overnight at 4^°^C. After removal of the underdrain, plates were washed (3 times with PBS-0.05% Tween 20 and 6 times with PBS) and developed with 3-amino-9-ethylcarbazole (AEC) substrate (BD Biosciences). The plates were washed thoroughly with distilled water and allowed to dry overnight. Spot-forming cells were subsequently enumerated in a blinded fashion using a dissection microscope. The number of toxin A- and B-specific IgG ASCs were expressed as a percentage of the total number of IgG antibody secreting cells. For analysis, the number of background spots from control wells (those containing unstimulated cells or lacking capture antigen) were subtracted from those coated with antigen.

### Culture of intestinal mucosa samples

To investigate antibody secretion by intestinal lamina propria cells, fresh mucosal samples (which were surplus to clinical requirements) were obtained from surgically-resected intestinal tissue of patients with ulcerative colitis or Crohn’s disease. Following removal of epithelial cells with EDTA, small pieces of mucosal samples were cultured in RPMI 1640 containing 10% fetal calf serum, 2mM L-glutamine and antibiotics for 24 hours, as previously described [13]. Supernatant samples were subsequently collected (after centrifugation to remove lamina propria cells) and stored at -80^0^C, prior to use in ELISA.

### Purification of C. difficile toxins

Toxins A and B were purified from supernatant samples of anaerobically cultured *C. difficile* VPI strain 10463, as previously described [15,16,17].

### Statistical analysis

Groups of patients were compared using two-tailed non-parametric tests (Spearman correlation, Kruskal-Wallis, Wilcoxon matched-pairs signed rank and Mann Whitney tests) and Fisher’s exact test. Data are expressed as median (range). Multiple serum samples were studied from many patients. Fluctuation in serum antibody concentrations in individual subjects over time was assessed using coefficient of variation. For comparative studies between groups, if more than one serum antibody concentration was determined, mean anti-toxin A and anti-toxin B antibody values were used per patient. A significance level of ≤0.05 was considered statistically significant.

## Results

The characteristics of the subjects in the four study groups are shown in Table 1, which also demonstrates that patients with *C. difficile*-associated diarrhoea were significantly older than those in the other three groups. In 16 of the 18 patients with cystic fibrosis, there was no history of *C. difficile* infection; 2 had a history of previous *C. difficile* infection; stool samples from a further 2 patients grew *C. difficile* and they were therefore deemed to be carriers. During the study period, patients in the cystic fibrosis group had significantly more hospital admissions than those in the other two patient groups (see table 1). At study enrolment, out of 18 of patients in the cystic fibrosis group, 15 and 3 patients were on 2 and 3 concurrent intravenous antibiotics, respectively. The most commonly prescribed antibiotics in descending order were Meropenem, Tobramycin, Amikacin, and Ceftazidime. In the inflammatory bowel disease group, 5 patients had no history of antibiotic usage within the 6 weeks prior to *C. difficile* infection. In the other 5 patients, two were on 2 types of intravenous antibiotics and one patient was on 3 antibiotics (antibiotics used included Co-amoxiclav, Gentamicin, Trimethoprim, Meropenem and Piperacillin and Tazobactam). In patients with *C. difficile*-associated diarrhoea, the most commonly prescribed antibiotics in descending order were Co-amoxiclav, Piperacillin and Tazobactam, Flucloxacillin, and Meropenem.

**Table 1:** Study subject characteristics and anti-toxin antibody responses.

	Healthy controls (n=19)	Patients with *C. difficile*-associated diarrhoea (n=53)	IBD patients with *C. difficile* infection (n=10)	Cystic fibrosis patients (n=18)
Age (yrs)	30 (22-39)	71 (21-98)*	34.5 (21-83)	27.5 (19-49)
Male/female	10/9	22/31	4/6	10/8
Diabetes mellitus	0	7	0	16
Liver disease	0	3	0	5
PEG/NG feeding	0	2	0	5
Immunosuppressive treatment (corticosteroid/other)	0	9/3	6/4	2/2^#^
PPI / H2 blocker	0	31	4	14
Pancreatic enzyme supplements	0	0	1	17
Number of hospital admissions over study period	0	3 (1-25)^a^	1 (1-5)^b^	4.5 (1-10)
Serum anti-toxin A Igg (EU)^§^	10.27 (0.01-59.51)^c^	17.74 (0.01-161.50)^d^	47.44 (0.01-182.40)	41.78 (1.62-159.4)^†^
Serum anti-toxin B Igg (EU)^§^	16.08 (0.01-70.68)^e^	38.57 (0.01-97.97)^f^	26.37 (1.62-93.08)	62.7 (12.17-89.12)^†^

* p<0.003 vs other groups

# post liver / lung transplantation

§ if more than one serum sample was studied per subject, the mean antibody concentration was used for analysis

† data from 16 patients with no history of *C. difficile* infection [mean values for the two patients with history of *C. difficile* infection: anti-toxin A: 109.5 (86.96-125.48) and 112.00 (84.55-129.71); anti-toxin B: 87.53 (68.34-96.95) and 61.31 (53.42-77.63)].

Comparison with cystic fibrosis group: ^a^p<0.05, ^b^p<0.01, ^c^p=0.004, ^d^p=0.02, ^e^ p = 0001, ^f^p=0.0073

IBD = inflammatory bowel disease; PEG = percutaneous endoscopic gastrostomy; NG = nasogastric, PPI = proton pump inhibitor

### Serum IgG levels

**Figure 1:**
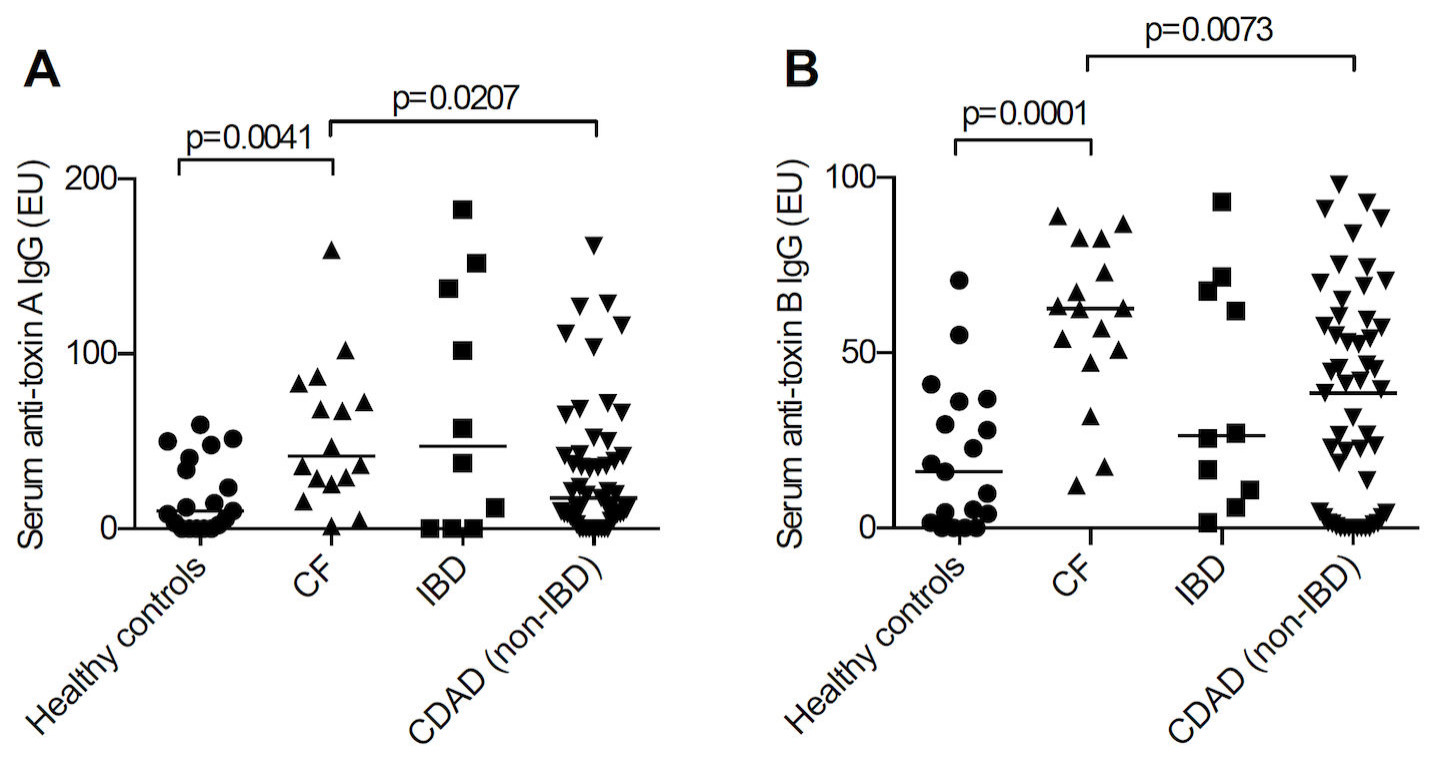
Serum anti-toxin IgG. Serum anti-toxin A (A) and anti-toxin B (A) IgG levels in healthy controls (n=19), patients with cystic fibrosis (CF; with no previous history of *C. difficile* infection; n=16), patients with inflammatory bowel disease (IBD) and *C. difficile* infection (n=10) and patients with *C. difficile*-associated diarrhoea (CDAD; n=53). If more than one serum sample was studied per subject, the mean antibody concentration was used for the calculation. Anti-toxin A and B levels in patients with cystic fibrosis were significantly higher than those observed in healthy controls and patients with *C. difficile*-associated diarrhoea.

The majority of serum samples from healthy controls had detectable anti-toxin IgG to toxin A (73.7%) and toxin B (78.9%). Concentrations of anti-toxin -A and -B IgG were significantly higher in patients with cystic fibrosis (with no previous history of *C. difficile* infection) than in healthy controls and patients with *C. difficile*-associated diarrhoea (Table 1 and Figure 1).

Compared with samples from the inflammatory bowel disease patients (n = 22), a significantly greater proportion of serum samples from the cystic fibrosis patients (n = 40) were positive for anti-toxin A IgG (97.5% vs 77.3%, p=0.0183) and anti-toxin B IgG (100% vs 77.3%, p=0.0041).

In paired comparisons, using all the samples collected, there was no correlation between serum anti-toxin A and anti-toxin B antibody levels in any of the patient groups.

### Serum anti-toxin IgG concentrations over time

**Figure 2:**
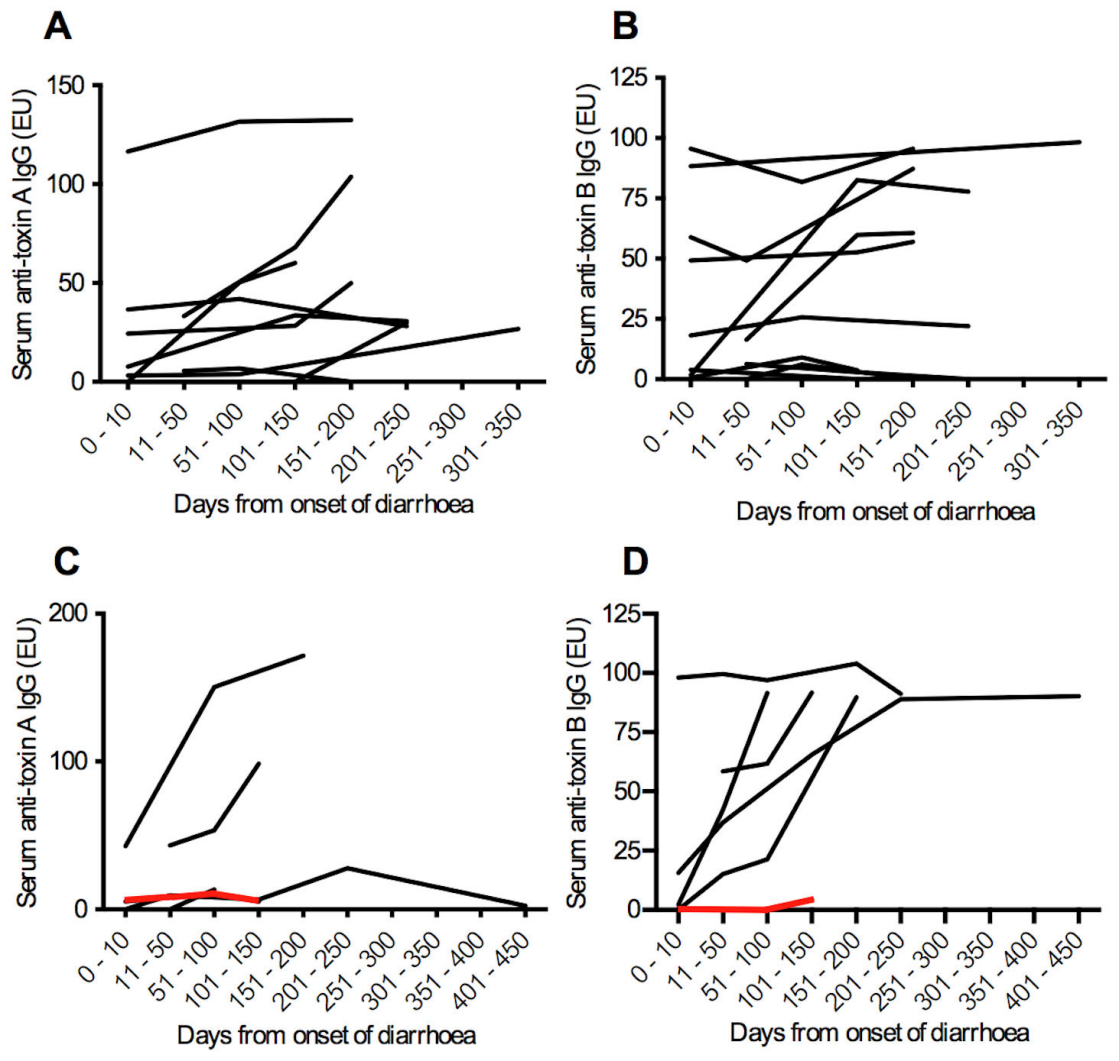
Serum anti-toxin IgG over time in patients with *C. difficile*-associated diarrhoea. (**A**, **B**) Serum anti-toxin A and B IgG levels respectively in patients with a single episode of *C. difficile* infection. (**C**, **D**) Anti-toxin A and B IgG levels respectively in patients with recurrent disease. The red line indicates a patient with 8 recurrences. Data are for patients from whom three or more serum samples were obtained.

In those patients with *C. difficile*-associated diarrhoea from whom 3 or more serum samples were obtained, serum anti-toxin A IgG and anti-toxin B IgG were seen to fluctuate over time. This is illustrated in Figure 2.

Within the patients with *C. difficile* associated diarrhoea, no significant difference in serum anti-toxin IgG was seen between those patients with single episode or recurrent disease (data not shown). However, in a patient with 8 episodes of *C. difficile*-associated diarrhoea either very low or undetectable anti-toxin IgG levels were observed (Figure 2). This patient went on to have treatment with intravenous pooled human immunoglobulin and had no further recurrences during the subsequent 12 month follow-up period.

**Figure 3:**
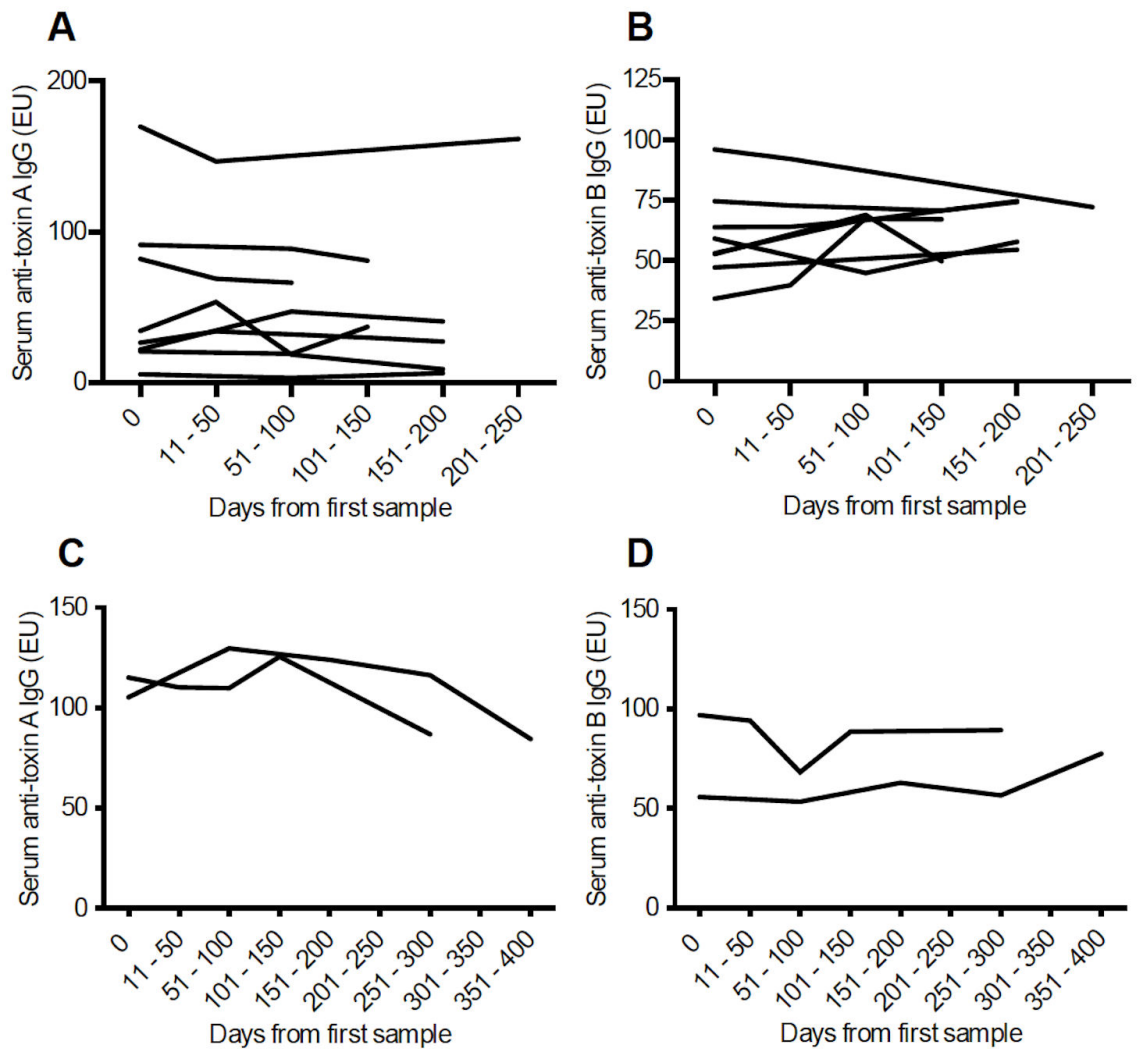
Serum anti-toxin IgG over time in patients with cystic fibrosis. (**A**, **B**) Serum anti-toxin A and B IgG levels respectively in patients without a history of *C. difficile* infection. (**C**, **D**) Anti-toxin A and B IgG levels respectively in patients with a history of *C. difficile* infection. Data are for patients from whom three or more serum samples were obtained.

**Figure 4:**
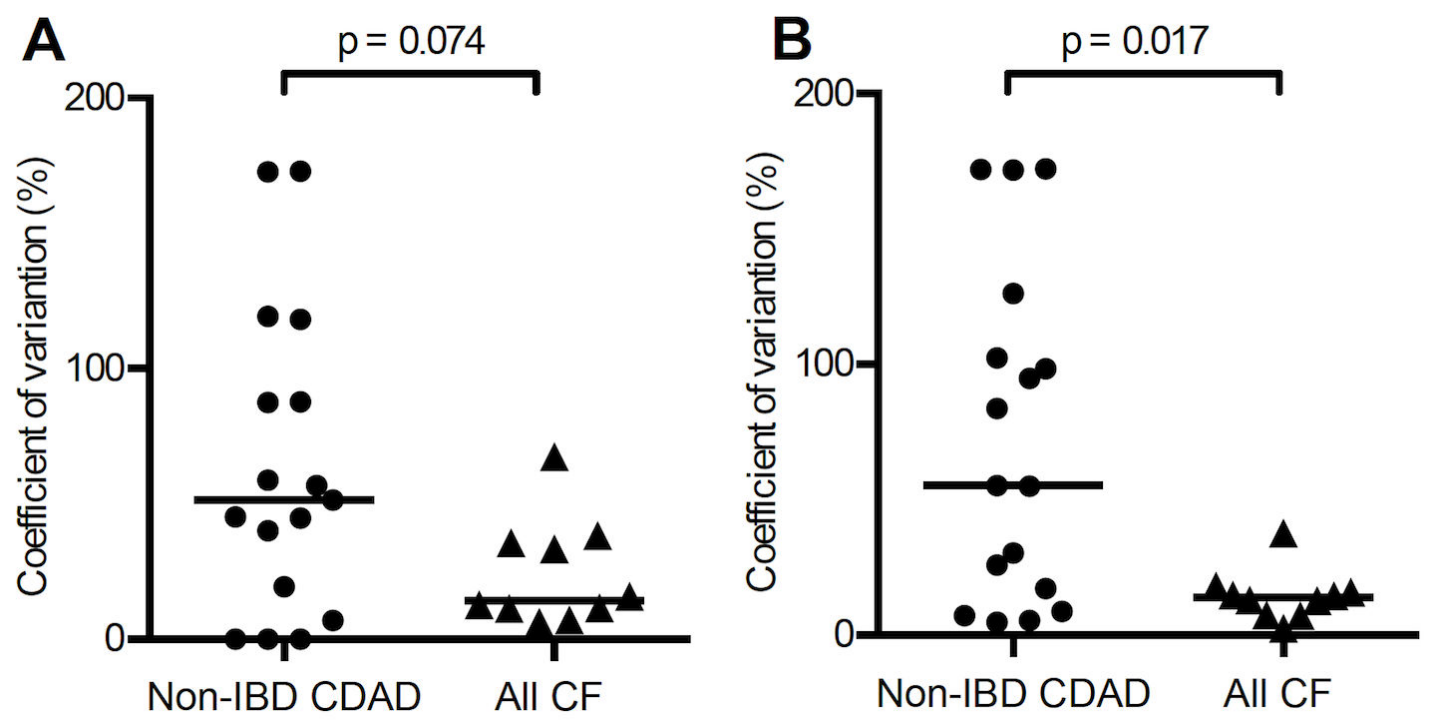
Stability of toxin-specific antibody responses over time. Coefficient of variation of (**A**) serum anti-toxin A and (**B**) anti-toxin B antibody concentrations in patients with non-IBD CDAD and cystic fibrosis (subjects with 3 or more serum antibody values). There is greater stability (ie less fluctuation) in toxin-specific antibody levels in patients with cystic fibrosis than those with non-IBD CDAD; this reaches statistical significance for anti-toxin B (p=0.017), but not anti-toxin A (p=0.074).

In those patients with cystic fibrosis, serum anti-toxin A and anti-toxin B IgG concentrations remained largely stable over time (Figure 3). A measure of stability over time was made by calculating the coefficient of variation for IgG levels in those patients with 3 or more samples. Significantly greater variation in serum anti-toxin B IgG concentrations were seen in patients with *C. difficile*-associated diarrhoea when compared with patients with cystic fibrosis and is shown in Figure 4 [anti-toxin A: 51.61% (0.0-173.0) vs 14.41% (6.31-67.42), p=0.074; anti-toxin B: 55.37% (4.73-172.4) vs 13.82% (2.45-37.78), p=0.017].

### Circulating C. difficile toxin A-specific antigen activated B cell responses

Peripheral blood mononuclear cells of cystic fibrosis patients without a history of diarrhoea [n=4; median age 23 yrs (20-26 yrs)] and non-CF patients with *C. difficile*-associated diarrhoea [n=20; median age 67 yrs (32-96 yrs), blood samples obtained within 10 days of the onset of diarrhoea] were exposed to fluorescently-labelled toxin A^488^ and labelled with antibodies to CD19 (pan B cell marker) and IgD (expression lost in switched memory B cells).

**Figure 5:**
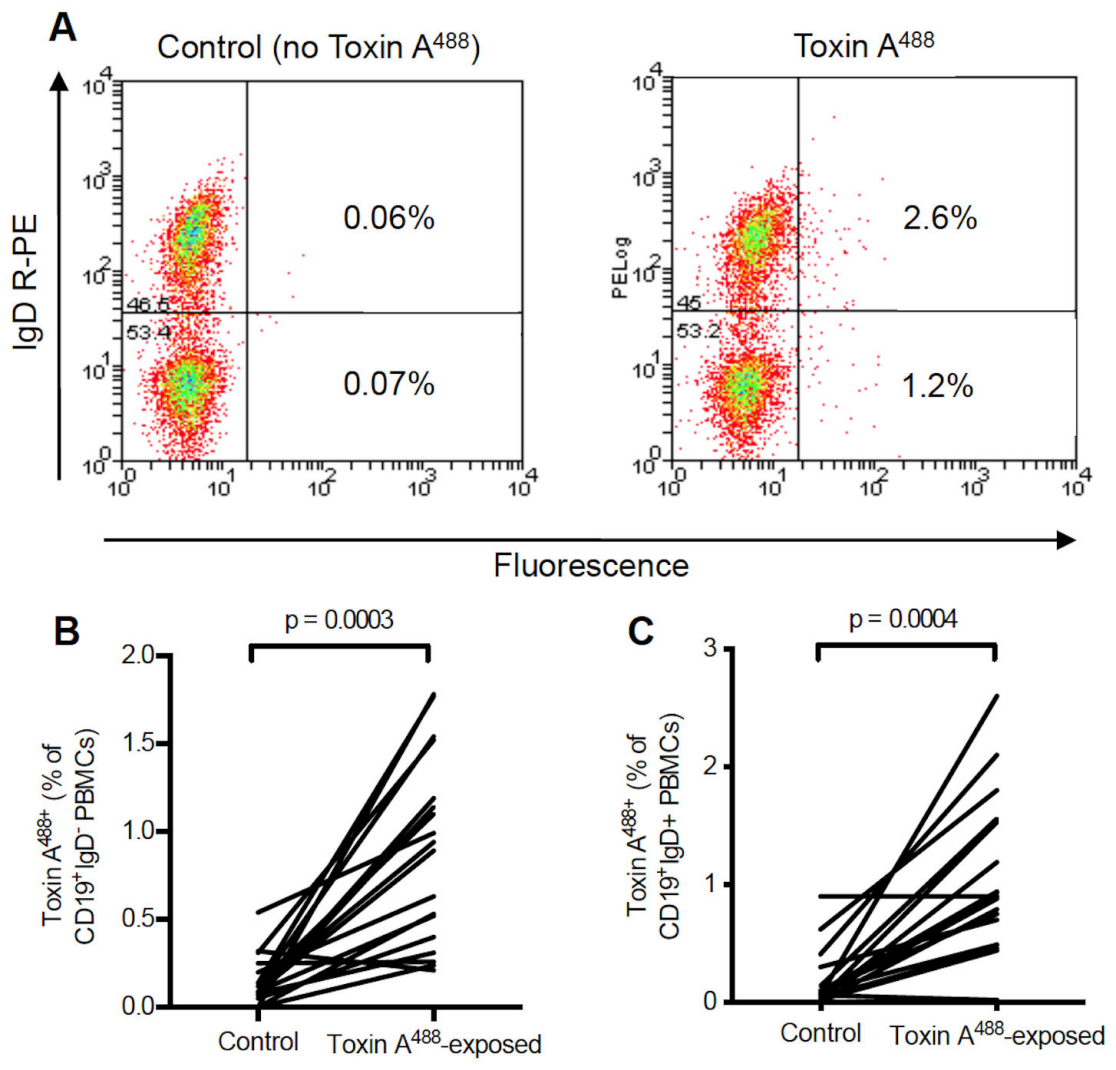
Circulating *C. difficile* toxin A-specific, antigen activated B cells. (**A**) Dot plots of CD19-positive peripheral blood mononuclear cells isolated (within 10 days of onset of symptoms) from a patient with *C. difficile* infection and incubated with either control buffer or toxin A^488^. IgD-negative and IgD-positive B cells that bound toxin A^488^ are represented by events in the right lower and upper quadrants, respectively. (**B**, **C**) Toxin A^488^ labelled events as a percentage of CD19-positive/IgD-negative (**B**) and CD19-positive/IgD-positive (**C**) events in patients with *C. difficile*-associated diarrhoea. When compared with control buffer, a significantly greater proportion of events were seen in both the CD19-positive/IgD-negative and CD19-positive/IgD-positive gates in peripheral blood mononuclear cells exposed to toxin A^488^.

In patients with *C. difficile*-associated diarrhoea, a significantly greater proportion of events were seen in the CD19-positive, IgD-negative gate in PBMCS exposed to toxin A^488^ [0.09% (0-0.54) versus 0.92% (0.09-1.78%); p<0.001 Wilcoxon matched-pairs], compared to control buffer. These results suggest that a small proportion of toxin A-specific, antigen-activated B cells can be detected in the peripheral circulation of patients soon after *C. difficile* infection (table 2; Figure 5). Compared to control buffer, a significantly greater proportion of toxin A^488^-specific events were seen in the CD19-positive/IgD-positive gates in patients with *C. difficile* associated diarrhoea.

**Table 2:** Toxin A-specific antigen-activated B cell frequencies by flow cytometry.

	Control CD19+ve/IgD-ve events (%)	Tox A^488^ CD19+ve/IgD-ve events (%)	Control CD19+ve/IgD+ve events (%)	Tox A^488^ CD19+ve/IgD+ve events (%)
Patients with *C. difficile*- associated diarrhoea (n=18) †	0.10 (0 - 0.54)	0.91* (0.21-1.78)	0.07 (0.01-0.9)	0.90* (0.02-2.60)
IBD patients with *C. difficile* infection (n=2)†	0, 0	1.26, 0.09	0.18, 0.21	1.24, 0.54
Cystic fibrosis patients, asymptomatic carriers of *C. difficile* (n=2)	0.08, 0	5.64, 1.14	0.04, 0.04	3.70, 1.42
Cystic fibrosis patients, stool negative for *C. difficile* (n=2)	0.08, 0.07	0.95, 0.45	0.06, 0.10	1.33, 0.86

* p<0.0005, vs relevant control

†Peripheral blood mononuclear cells were studied within 10 days of onset of diarrhoea.

In the two cystic fibrosis patients who were asymptomatic carries of *C. difficile*, >1% (up to 5.64%) of toxin A^488^-specific events were seen in the above gates (table 2).

### Circulating memory B cell responses to C. difficile toxins

Following mitogen-induced differentiation of memory B cells, toxin A- and B-specific antibody secreting cells were enumerated using ELISpot assays and expressed as a percentage of total IgG-secreting cells.

In initial tests of polyclonal activation, the concentration of human IgG secreted into PBMC cell culture supernatant samples (34 samples from 19 patients) was significantly higher following mitogen stimulation compared to cells cultured in control medium [median 0.026 µg/ml (range 0.0-6.454) vs 0.465 µg/ml (0.001-7.172); p <0.0001). Anti-toxin A and B IgG was detectable in 12% and 26.2% of supernatant samples of mitogen-stimulated peripheral blood mononuclear cells, respectively. Toxin A-specific (66.7%) and B-specific (100%) antibody secreting cells were detected in their respective ELISpot assays following polyclonal stimulation of peripheral blood mononuclear cells.

**Figure 6:**
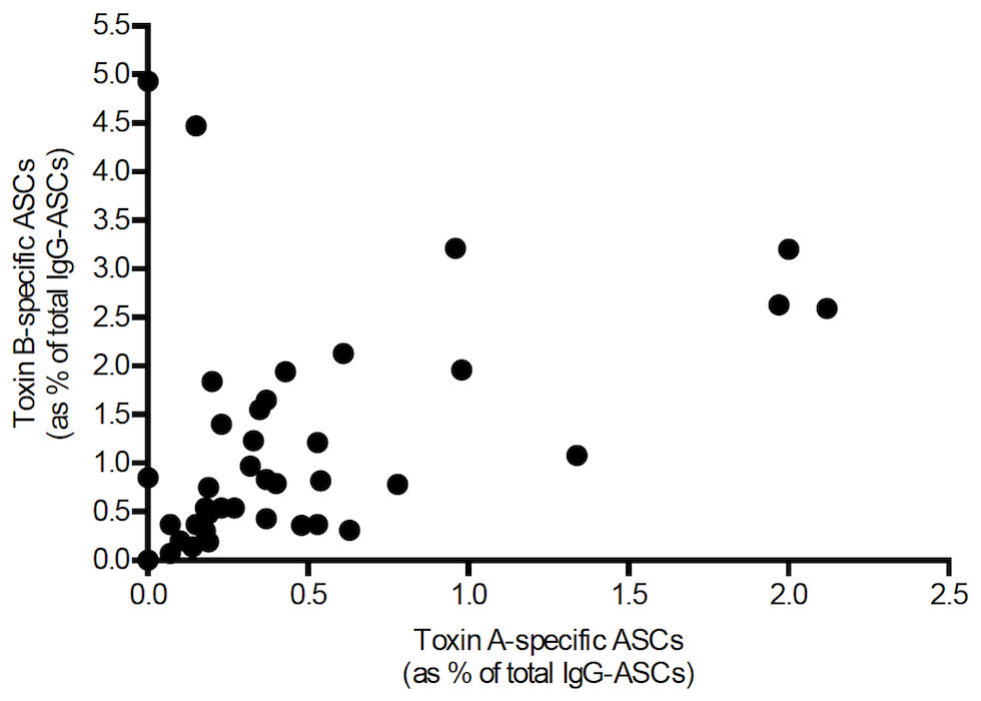
Correlation of toxin A- and B-specific ASC frequencies in patients with *C. difficile*-associated diarrhoea. Peripheral blood mononuclear cells from 39 blood samples, collected at varying time intervals after onset of *C. difficile*-associated diarrhoea in 16 patients (median 181 days after onset of diarrhoea, range 81-415). There was significant correlation (p=0.0035) between the frequency of toxin A- and toxin B-specific IgG secreting cells (as percentage of total IgG-secreting antibody secretory cells).

Peripheral blood mononuclear cells were isolated from 39 blood samples, collected at varying time intervals after onset of *C. difficile*-associated diarrhoea in 16 patients [samples obtained at median 181 (range 81-415) days after the onset of symptoms]. There was significant correlation (p=0.0035) between the frequency of toxin A- and toxin B-specific antibody secreting cells (Figure 6).

**Figure 7:**
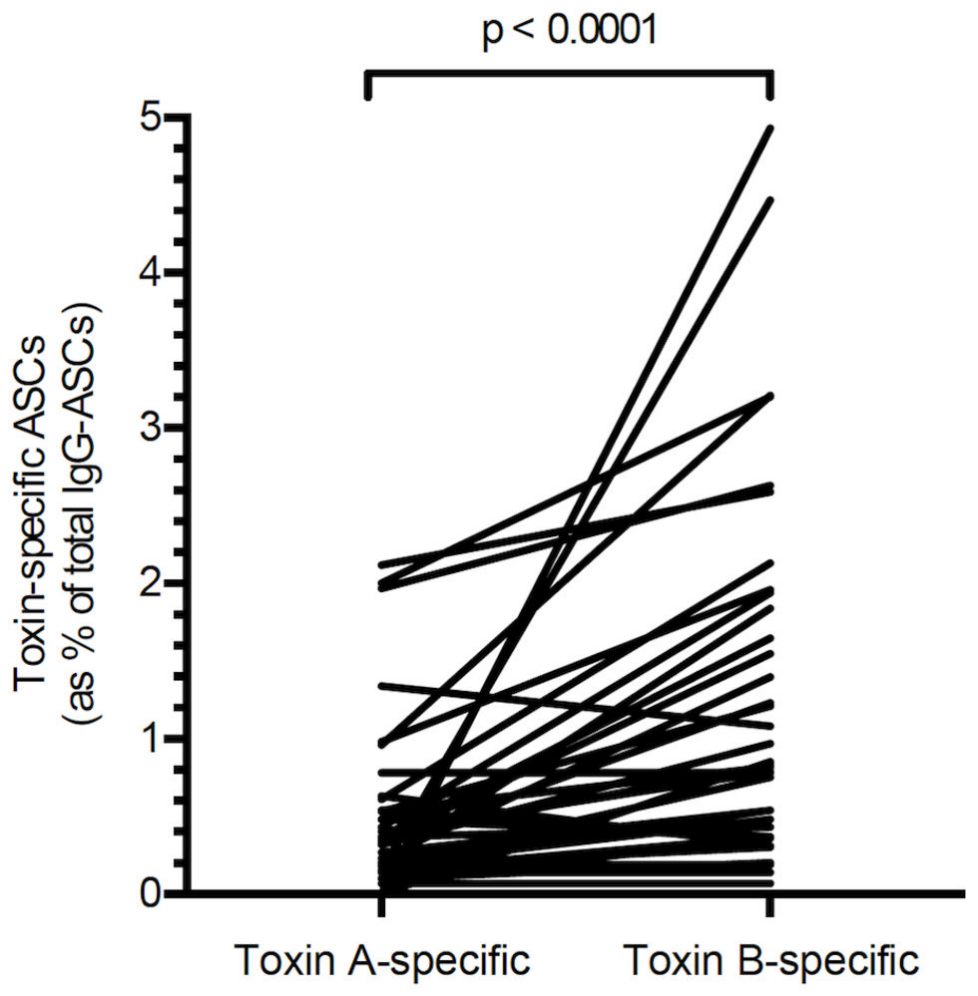
Toxin A and B-specific ASC frequencies in patients with *C. difficile*-associated diarrhoea. Peripheral blood mononuclear cells from 39 blood samples, collected at varying time intervals after onset of *C. difficile*-associated diarrhoea in 16 patients. A significantly higher proportion of toxin B-specific antibody secretory cells (than toxin A-specific antibody secretory cells) was detected [0.82 (0 - 4.93) % vs 0.33 (0 - 2.12) %; p<0.0001].

In the same samples, a significantly greater proportion of toxin B-specific, than toxin A-specific memory B cells were detected [0.82% (0 - 4.93) vs 0.33% (0 - 2.12); p<0.0001] (Figure 7).

**Figure 8:**
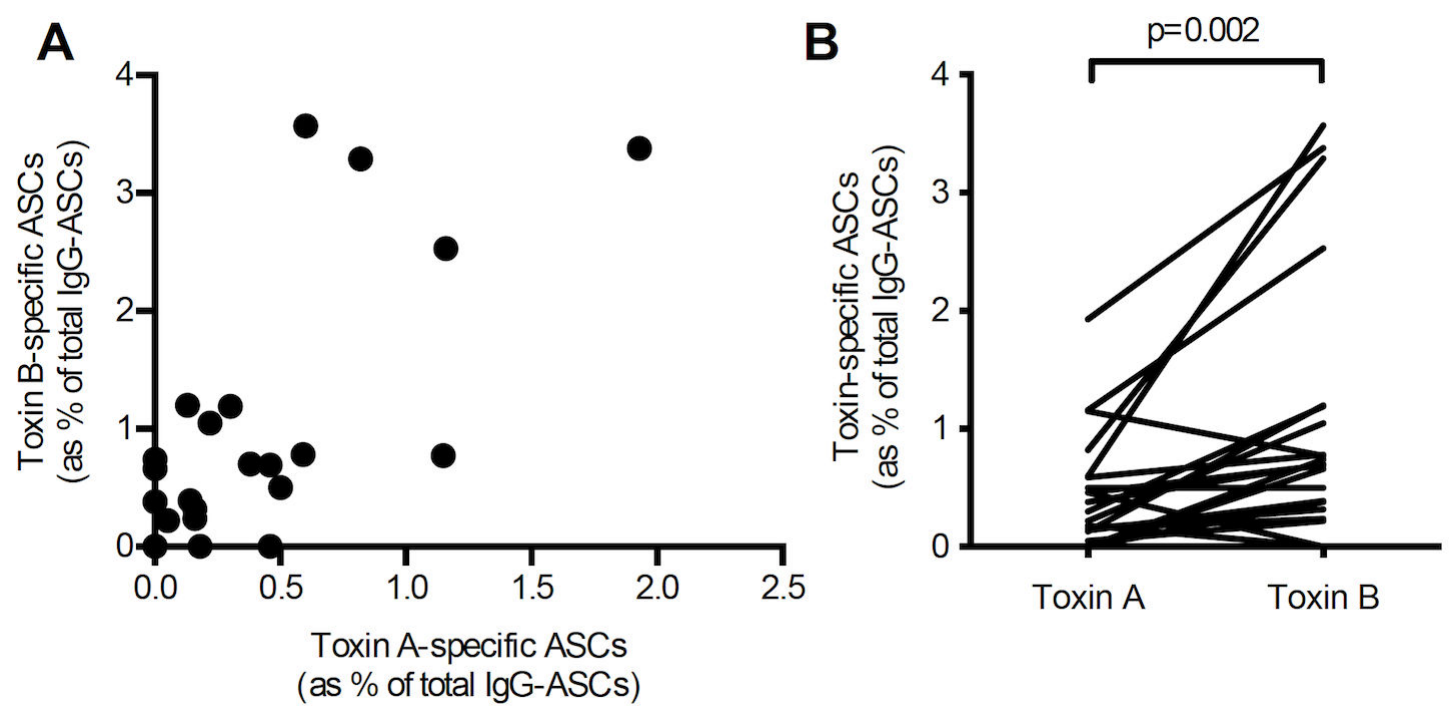
Toxin A and B-specific ASC frequencies in patients with cystic fibrosis. Peripheral blood mononuclear cells from 24 blood samples, collected at varying time intervals, from 13 patients with cystic fibrosis (with no previous history of *C. difficile* infection) [samples obtained over median 161.5 (range 42-244) days]. There was significant correlation (p=0.0017) between toxin A- and toxin B-specific IgG antibody secreting cells (**A**) with significantly higher proportions of the latter [0.67 (0 - 3.57) %) vs 0.20 (0 - 1.93) %; p=0.002] (**B**).

Similar findings were observed in samples obtained from four patients with inflammatory bowel disease and *C. difficile* infection and also in samples from patients with cystic fibrosis (without a history of *C. difficile* infection) [0.67% (0 - 3.57) vs 0.20% (0 - 1.93); p=0.002] (Figure 8).

**Figure 9:**
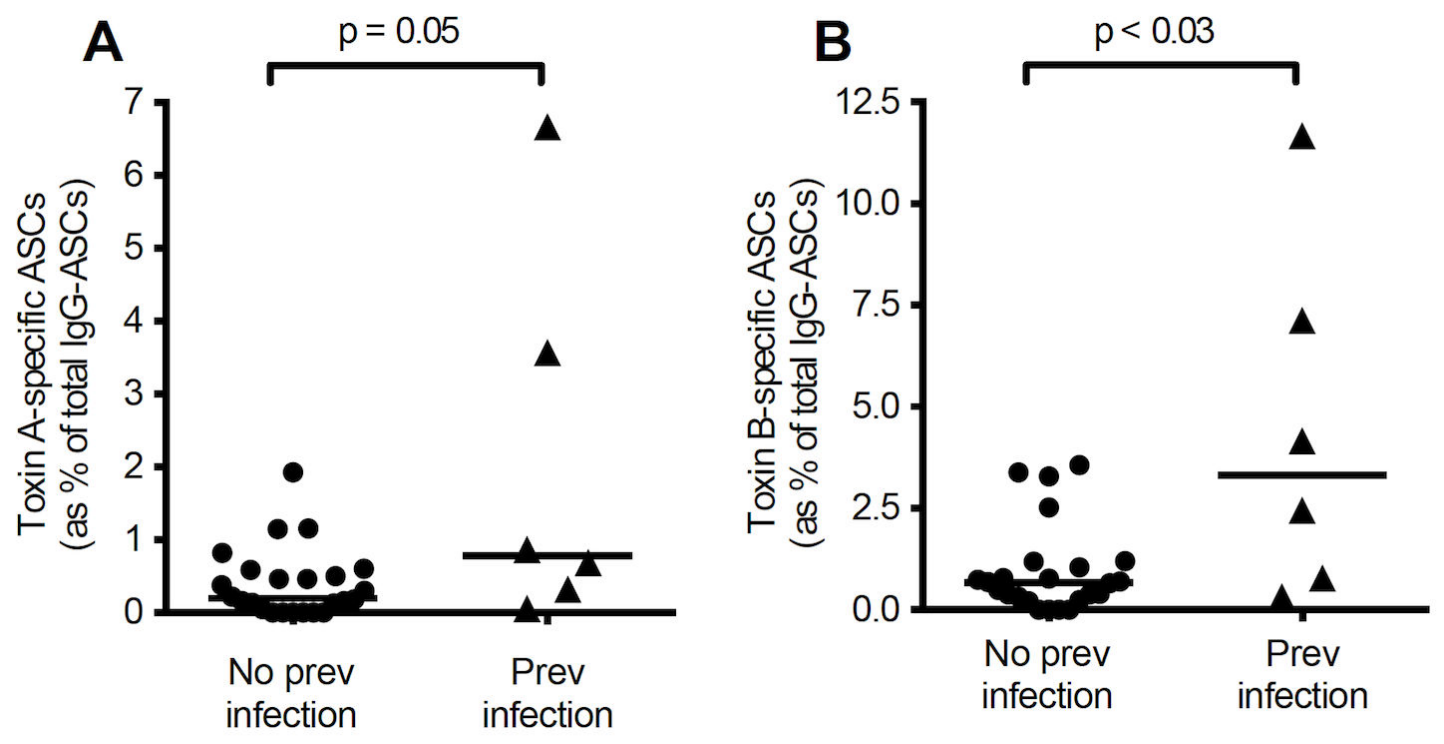
Toxin A- and B-specific ASCs in patients with cystic fibrosis with and without previous *C. difficile*. Peripheral blood mononuclear cells were isolated from a total of 6 and 24 samples collected at varying time intervals from patients with cystic fibrosis with (n=2) and without (n=13) previous history of *C. difficile* infection, respectively. The frequencies of toxin A- and B-specific antibody secreting cells were significantly higher in those patients with previous *C. difficile* infection than those without. (**A**) toxin A-specific ASCs (p=0.05); (**B**) toxin B-specific ASCs (p=0.026).

In circulating cells collected at 6 different time points from 2 patients with cystic fibrosis and a history of *C. difficile* infection, there were significantly higher toxin A- and toxin B-specific antibody secreting cells, compared to cells isolated from 13 cystic fibrosis patients with no previous history of *C. difficile* infection [toxin A: 0.20% (0.0-1.93) vs 0.78% (0.06-6.67), p=0.05; and toxin B: 0.675% (0.0-3.57) vs 3.315% (0.32-11.67), p<0.03] (Figure 9). The highest proportion of toxin A-specific (6.67%) and toxin B-specific (11.67%) antibody secreting cells was observed in one of the patients with cystic fibrosis and a history of *C. difficile* infection, (sample obtained 121 days after the onset of *C. difficile*-associated diarrhoea).

### Antibody and memory B cell responses to toxins A and B in individual subjects

There was no correlation between serum anti-toxin IgG and memory B cell frequencies in samples obtained from patients with *C. difficile*-associated diarrhoea, inflammatory bowel disease (with *C. difficile* infection) and those with cystic fibrosis.

**Figure 10:**
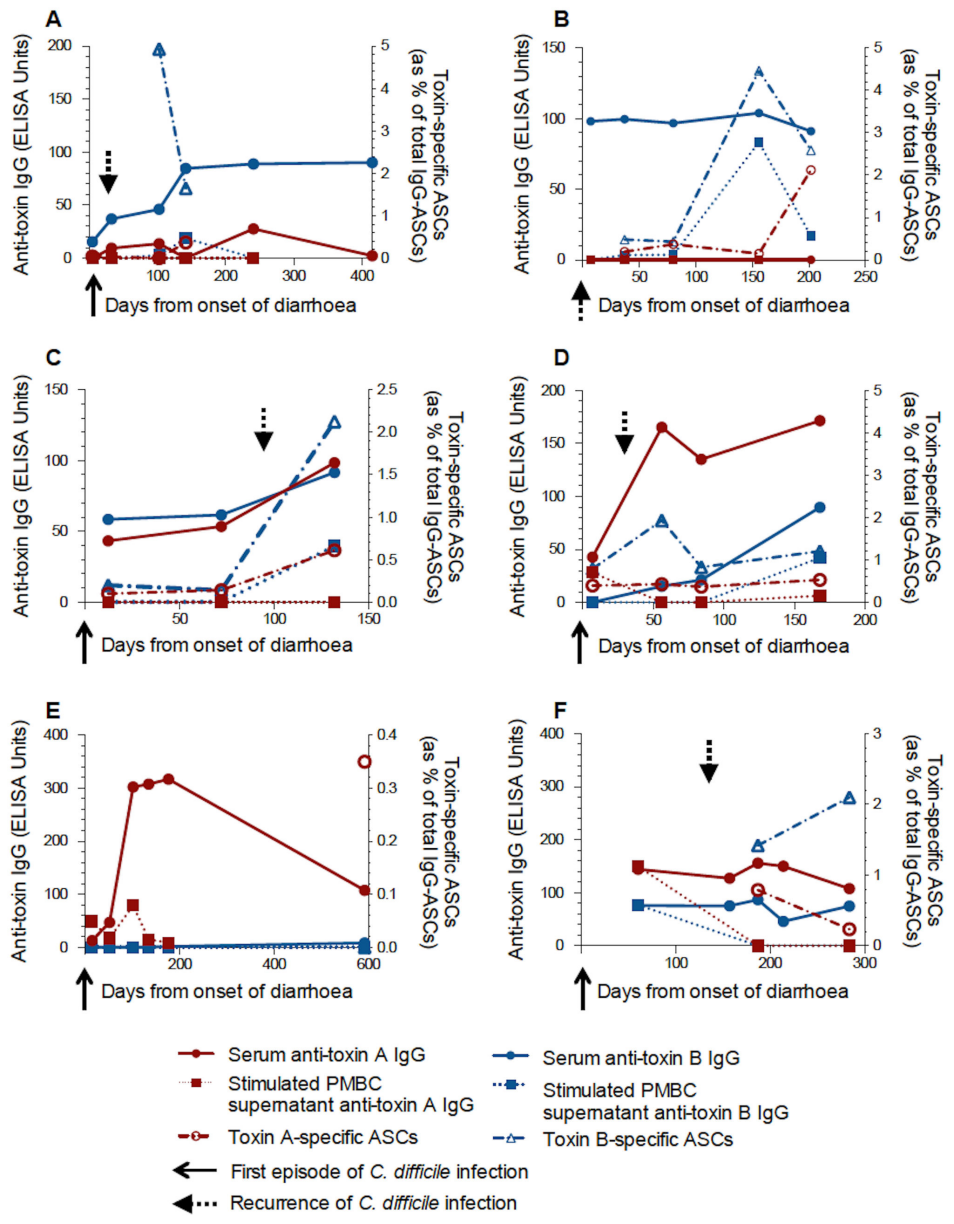
Toxin-specific antibody and memory B cell responses in non-IBD and IBD patients. Case studies (A–F) showing toxin A- and toxin B-specific serum antibody levels and memory ASC frequencies in non-IBD CDAD and IBD-CDAD patients. (**A**) 67 yr female, 1st sample 5d after onset of diarrhoea associated with 1st episode of *C. difficile* infection. Recurrence of infection after 26d (dashed arrow) with no further episodes over 21mths. (**B**) 62 yr female, 1^st^ sample 7d after 2nd episode of *C. difficile* infection (solid arrow). No recurrences over 14mths. (**C**) 71 yr female, 1st sample 12d after onset of diarrhoea associated with 1st episode of severe *C. difficile* infection (solid arrow). Second episode of infection (dashed arrow) after 94d but no further episodes over subsequent 15mths. (**D**) 62 yr female, 1st sample 7d after onset of diarrhoea associated with 1st episode of severe *C. difficile* infection (solid arrow). Recurrence at 30d (dashed arrow). No further episodes of infection over subsequent 13mths. (**E**) 32 yr pregnant female, recent diagnosis of ulcerative colitis. Hospital admission for intravenous corticosteroids 17d prior to 1st sample. Two initial stool samples negative for *C. difficile* toxin. Four days before first blood sample, positive stool *C. difficile* toxin test (solid arrow). (**F**) 28 yr female with ulcerative colitis and recurrent *C. difficile* infection. First sample during 3^rd^ episode of *C. difficile* infection (1st episode occurred 60d previously – solid arrow). Fourth episode of *C. difficile* infection (dashed arrow) at 135d. In intervening period, patient was taking corticosteroids, ciclosporin and azathioprine.

**Figure 11:**
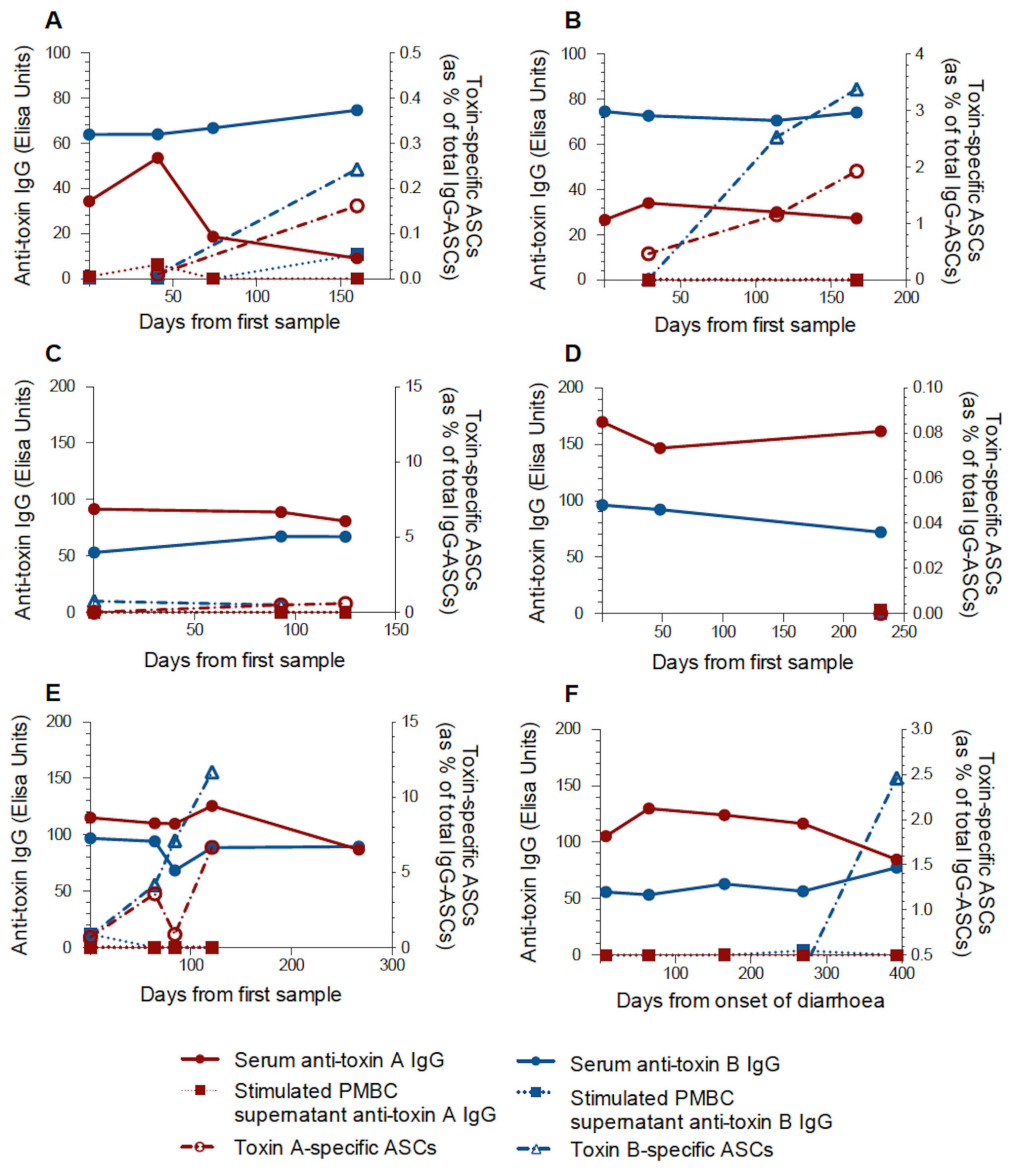
Toxin-specific antibody and memory B cell responses in cystic fibrosis patients. Case studies (A–F) showing toxin A- and toxin B-specific serum antibody levels and memory ASC frequencies in patients with cystic fibrosis. (**A**) 26 yr male with insulin-dependent diabetes mellitus, previous fundoplication and gastrostomy. Colonised with *C. difficile* at recruitment. No history of diarrhoea during study period and subsequent 17mths. (**B**) 24 yr male with insulin-dependent diabetes mellitus. Colonised with *C. difficile* at recruitment. (**C**) 32 yr male with insulin-dependent diabetes mellitus. Not colonised with *C. difficile* at recruitment. No history of diarrhoea during the study or follow-up period. (**D**) 24 yr male with insulin-dependent diabetes mellitus, previous bilateral lung transplantation 3d after first sample. Subsequently taking prednisolone, azathioprine, tacrolimus and cotrimoxazole. (**E**) 34 yr female with insulin-dependent diabetes mellitus. Overnight feeding via jejunostomy. *C. difficile* infection with diarrhoea 10mths prior to first sample. No recurrence of *C. difficile* infection during the study or subsequent 9 month follow-up period. (**F**) 28 yr male recruited during episode of clinical *C. difficile* associated diarrhoea. No recurrence of *C. difficile* infection during the study period or 14mth follow-up period.

Illustrative examples are shown in Figures 10 and 11. In some patients in the *C. difficile*-associated diarrhoea and inflammatory bowel disease (with *C. difficile* infection) groups, serum IgG responses were not seen to either toxin A or B. In the majority of the cystic fibrosis patients (17 of 18), serum antibody responses were seen to both toxins.

In three patients (Figure 10 A, C, D) with *C. difficile*-associated diarrhoea, serum anti-toxin IgG levels rose following a second episode of infection and no further clinical recurrence was observed.

### Secretion of anti-toxin IgG by intestinal lamina propria cells

In a group of patients with inflammatory bowel disease and no history of *C. difficile* infection, secretion of toxin-specific IgG antibodies by intestinal lamina propria cells was studied in supernatant samples of cultured pieces of small and large intestinal mucosa obtained from operation resection specimens.

Supernatant samples were diluted 1:4 and ELISA Units (EU) determined from OD_405_ values using a standard curve established from serial dilutions of pooled serum samples with high anti-toxin antibody titres.

In 2 (of 5) supernatant samples of mucosal tissue from patients with ulcerative colitis and 3 (of 8) with colonic Crohn’s disease, anti-toxin B IgG was detected, but anti-toxin A IgG was undetectable in all.

In cultures of 7 samples of small intestine from patients with Crohn’s disease, anti-toxin A and anti-toxin B IgG were detected in 2 and 7 respectively.

Anti-toxin A and B IgG antibodies were detected in supernatant samples of cultured inflamed colonic mucosa from a patient with *C. difficile*-associated pseudomembranous colitis undergoing colectomy.

## Discussion

Following initial exposure to a pathogen or its products, protection by the humoral immune system is mediated by long-lived plasma cell-derived antibodies and long-lived memory B cells [18]. Our studies have investigated both types of IgG-mediated humoral immune responses to *C. difficile* toxins in patients with *C. difficile*-associated diarrhoea, IBD patients with *C. difficile* infection and cystic fibrosis patients, the majority of which had no previous history of *C. difficile* infection. Serum anti-toxin IgG was also quantified in a separate groups of healthy subjects.

Consistent with previous studies [19,20], the majority of the healthy controls in our studies had detectable anti-toxin A and anti-toxin B antibodies in their serum samples. This implies that asymptomatic exposure to toxigenic *C. difficile* is common and may be due to transient colonisation. Notably, significantly higher anti-toxin antibody concentrations in patients with cystic fibrosis (with no previous history of *C. difficile* infection) are likely to be due to more frequent contact with the toxins secreted by *C. difficile* that colonises the large intestine following antibiotic-mediated disruption of the protective resident microflora, especially following admission to hospital [21]. Indeed, in contrast to patients with inflammatory bowel disease, all the cystic fibrosis patients were on 2 or 3 intravenous antibiotics at the time of recruitment to the study. The cystic fibrosis patients also had additional risk factors for *C. difficile* colonization/infection including tube feeding, the use of proton pump inhibitors and H2 blockers [3,4]. Frequent exposure to the toxins may also explain maintenance of steady serum anti-toxin antibody concentrations in patients with cystic fibrosis. These circulating antibodies to toxins A and B are believed to provide protection against the development of disease [3,5,6,20], which may be best illustrated in the two asymptomatic carriers of toxigenic *C. difficile*. The development of *C. difficile* infection in two cystic fibrosis patients suggest that immune-mediated protection can be overcome and may reflect tilting of the balance in favour of the pathogen. Such “imbalance” may occur due to increased production of toxins A and B, for example following infection with the NAP1 strain [22], which has recently been reported to be associated more frequently with *C. difficile* infection (than colonization) [3]. High luminal concentrations of the toxins may overcome IgG and other (IgA, mucus) forms of protection, leading to enhanced susceptibility of host intestinal epithelial and lamina propria cells [15,23]. Whilst firm conclusions cannot be drawn because of the small number of subjects studied, cystic fibrosis patients with previous *C. difficile* infection appeared to have an enhanced memory B cell response and serum anti-toxin antibody levels above the mean for the rest of the group. Such heightened response may protect against recurrence.

Serum anti-toxin A and anti-toxin B antibody concentrations in patients with *C. difficile*-associated diarrhoea were significantly lower than in cystic fibrosis patients. Patients in the former group were also significantly older, which may explain the inability of these patients to mount as strong a humoral immune response as those younger patients with cystic fibrosis. Since many patients with *C. difficile* infection had fluctuating anti-toxin antibody levels, our studies raise the possibility that *C. difficile* toxin-specific long-lived plasma cell numbers and/or function may not be sustained over time. This could be a general feature of humoral immune responses to bacterial toxins as, in contrast to viral antigens, antibody responses to tetanus and diphtheria toxins have been reported to wane more quickly [24]. Moreover, studies of the aged human immune response (termed ‘immune senescence’) document qualitative and quantitative decline in T and B lymphocyte responses to a range of microbes and antigens. Such changes could be due in part to impaired T cell help for B cell IgG production, diminishing lymphocyte clonal repertoire diversity (polyclonal to oligoclonal) and microenvironmental factors in lymphoid tissue and bone marrow [25,26]. Such information is relevant in efforts to induce and sustain protective immunity against *C. difficile* infection via improved vaccination strategies.

Whilst concentrations of serum IgG antibodies to toxin A and toxin B did not differ between cystic fibrosis and inflammatory bowel disease patients, greater heterogeneity in antibody levels was seen in the latter group, as illustrated by the absence of detectable antibody in some serum samples. Significantly fewer hospital admissions in the inflammatory bowel disease group suggest that such heterogeneity could be due to less frequent exposure to *C. difficile* in the hospital environment. Further studies with larger groups of patients are required to determine whether patients with inflammatory bowel disease are at greater risk of *C. difficile* infection than those with cystic fibrosis. Of note, patients with long-standing diagnoses of ulcerative colitis and Crohn’s disease in clinical remission without recent exposure to antibiotics, immunomodulatory drugs or recent hospitalisation, demonstrate an increased frequency of detectable faecal *C. difficile* when compared with healthy adults [27]. However, it remains to be seen whether carriage of this bacterium in such patients ultimately translates into increased risk of relapse and will require larger numbers of subjects and longer-term follow-up. Further studies are also required to determine the impact of known risk factors for *C. difficile* acquisition including antibiotics and immunomodulatory drugs in relation to carriage rates in the IBD population [27].

Our studies in which cultures of some intestinal mucosal samples affected by inflammatory bowel disease secreted antibodies to *C. difficile* toxins suggest that exposure to toxigenic *C. difficile* is not uncommon in such patients. These patients were not thought to have had clinical *C. difficile* infection, implying asymptomatic exposure to toxigenic *C. difficile*, with protection mediated by anti-toxin antibodies secreted by plasma cells in the mucosa. It will also be of interest to determine whether the antibody producing cells in the mucosa of patients with inflammatory bowel disease represent long-lived plasma cells.

Impaired mucosal regulatory T cell function has been implicated in the pathogenesis of inflammatory bowel disease [28] but the effect of *C. difficile* infection on T cell function in patients with ulcerative colitis and Crohn’s disease remains to be determined. It is possible that *C. difficile* toxin-induced cytokine expression in macrophages [29] may lead to resistance of effector T cells to suppression by regulatory T cells and thereby lead to exacerbation of mucosal inflammation in inflammatory bowel disease. Changes in the resident microbial flora associated with *C. difficile* infection may also adversely influence mucosal regulatory T cells function.

In peripheral blood mononuclear cells, following differentiation of memory B cells, there was significant correlation between toxin A- and toxin B-specific antibody secreting cells (with greater proportions of the latter). However, there was lack of correlation between toxin A- or toxin B-specific serum antibody levels and the relevant memory B cell responses. Such lack of correlation between serum antibody concentrations (derived from long-lived plasma cells) and circulating memory B cell numbers has also been reported for other antigens [24] and likely reflects independent regulation of the development of the relevant cells in germinal centres [18].

The findings of toxin A488+ve CD19-positive/IgD-negative and CD19-positive/IgD-positive cells are consistent with the presence of switched (IgD-negative) and unswitched (IgD-positive), toxin A-specific memory B cells. They also reflect heterogeneity in the generation of memory B cell populations [30]. However, the functional contributions of these distinct subpopulations of cells in host protection remains to be determined. More detailed phenotypic studies aimed at characterising human memory B cell subsets in patients with *C. difficile* infection may help identify B cell signatures associated with disease severity, prognosis and response to treatment.

In conclusion, our studies provide further evidence for the importance of the humoral immune response to toxins A and B in protection against *C. difficile* infection. The impaired ability to generate strong and sustained toxin-specific humoral immune responses, via long-lived plasma cells and long-lived memory B cells, may increase susceptibility of older individuals to *C. difficile* infection and warrants further investigation of the role of immune senescence.

Overall, our data suggest that serum anti-toxin antibodies may underestimate the breadth of humoral immunity in relation to *C. difficile* infection and strongly support the use of toxin-specific B memory cell analyses to complement serological studies. The antigen-specific flow cytometric and ELISpot techniques described in this report could find utility in the development of an effective novel vaccine against *C. difficile* infection that induces long-lived protection.
